# Cigarette smoking and thinning of the brain’s cortex

**DOI:** 10.1038/mp.2014.187

**Published:** 2015-02-10

**Authors:** S Karama, S Ducharme, J Corley, F Chouinard-Decorte, J M Starr, J M Wardlaw, M E Bastin, I J Deary

**Affiliations:** 1 Department of Neurology and Neurosurgery, McConnell Brain Imaging Center, Montreal Neurological Institute, McGill University, Montreal, QC, Canada; 2 Department of Psychiatry, Douglas Mental Health University Institute, McGill University, Verdun, QC, Canada; 3 Department of Psychiatry, McGill University Health Centre, Montreal, QC, Canada; 4 Department of Psychiatry and Neurology, Massachusetts General Hospital, Harvard Medical School, Boston, MA, USA; 5 Department of Neurology, McLean Hospital, Harvard Medical School, Belmont, MA, USA; 6 Department of Psychology, University of Edinburgh, Edinburgh, UK; 7 Centre for Cognitive Ageing and Cognitive Epidemiology, University of Edinburgh, Edinburgh, UK; 8 Department of Psychology, Alzheimer Scotland Dementia Research Centre, University of Edinburgh, Edinburgh, UK; 9 Department of Radiology, Division of Neuroimaging Sciences, Brain Research Imaging Centre, University of Edinburgh, Edinburgh, UK

## Abstract

Cigarette smoking is associated with cognitive decline and dementia, but the extent of the association between smoking and structural brain changes remains unclear. Importantly, it is unknown whether smoking-related brain changes are reversible after smoking cessation. We analyzed data on 504 subjects with recall of lifetime smoking data and a structural brain magnetic resonance imaging at age 73 years from which measures of cortical thickness were extracted. Multiple regression analyses were performed controlling for gender and exact age at scanning. To determine dose–response relationships, the association between smoking pack-years and cortical thickness was tested and then repeated, while controlling for a comprehensive list of covariates including, among others, cognitive ability before starting smoking. Further, we tested associations between cortical thickness and number of years since last cigarette, while controlling for lifetime smoking. There was a diffuse dose-dependent negative association between smoking and cortical thickness. Some negative dose-dependent cortical associations persisted after controlling for all covariates. Accounting for total amount of lifetime smoking, the cortex of subjects who stopped smoking seems to have partially recovered for each year without smoking. However, it took ~25 years for complete cortical recovery in affected areas for those at the mean pack-years value in this sample. As the cortex thins with normal aging, our data suggest that smoking is associated with diffuse accelerated cortical thinning, a biomarker of cognitive decline in adults. Although partial recovery appears possible, it can be a long process.

## Introduction

Cigarette smoking has well-documented associations with numerous negative health outcomes including possible effects on the brain.^1^ Evidence suggests that smokers have, on average, slightly poorer global cognitive functioning in later life, as well as lower mean scores on several cognitive domains such as cognitive flexibility and memory.^2^ Longitudinal studies and meta-analyses report that smoking is associated with an increased risk of dementia^3^ and it is estimated that nearly 14% of Alzheimer’s disease cases worldwide could be attributable to smoking.^3^

In keeping with this, cigarette smoking has been linked to regional brain atrophy and reduced cortical volume in a few cortical regions.^4, 5, 6, 7, 8, 9, 10^ Recently, two studies reported associations between smoking and the brain’s cortex, using cortical thickness, a metric viewed as a proxy marker of cortical cytoarchitecture integrity and considered to be more sensitive to neurodegenerative processes than cortical volume.^11^ Kuhn *et al.*,^12^ comparing 22 smokers with 21 controls, and Durazzo *et al.*,^11^ comparing 43 smokers with 33 non-smokers with alcohol dependence, reported smoking-related cortical thinning in a few isolated cortical regions. However, larger samples may be required to detect local cortical thickness effects^13^ in whole-brain analyses, raising the possibility of type 2 statistical error in these studies.

Crucially, it is not known whether some of the possible effects of smoking on the brain are reversible. This is important given the unprecedented degree of aging of the world’s human population, the fact that many people continue to smoke into old age^2^ and the possibility that stopping early enough may help toward delaying dementia if it leads to a reversal of cortical thinning effects of smoking.

Here we examine the association between smoking and brain cortical thickness in the Lothian Birth Cohort 1936 (LBC1936).^14^ This group of community-dwelling individuals has undergone brain magnetic resonance imaging (MRI) at ~73 years of age and provided data on lifetime history of smoking, a valid measure of tobacco use.^15^ Many potential confounding variables are available on this cohort including a measure of cognitive ability at 11 years of age (that is, before beginning smoking). This is useful given previously reported positive associations between cognitive ability across the life course and cortical thickness in old age,^16^ negative associations between cognitive ability and likelihood of smoking, and positive associations between cognitive ability and probability of quitting smoking.^2^ Although one of the goals here was to reassess the extent of smoking-related cortical thinning by capitalizing on the increased power provided by the large brain imaging LBC1936 cohort, an important aim was to evaluate the degree of potential reversibility of smoking-related cortical thinning. In light of accruing evidence of brain plasticity in adults^17^ and the observed reversal of risk for some health outcomes after smoking cessation,^18^ we predicted at least a partial reversal of smoking-related cortical thinning in those who had stopped smoking.

## Materials and methods

### Sample

As members of the LBC1936, most participants had taken part in the Scottish Mental Survey of 1947 in which almost all Scottish school children born in 1936 and attending school in June 1947 were cognitively tested. In 2007, during the second Wave of the LBC1936 study, 866 surviving members from the Edinburgh area, who were able and willing to undergo a set of cognitive and medical tests,^14^ were invited to have brain MRI scans.^19^ From these, 666 underwent brain imaging including high-resolution structural T1-weighted MRI scanning used for cortical thickness estimation (see MRI acquisition section for scanning details).

**Figure 1:**
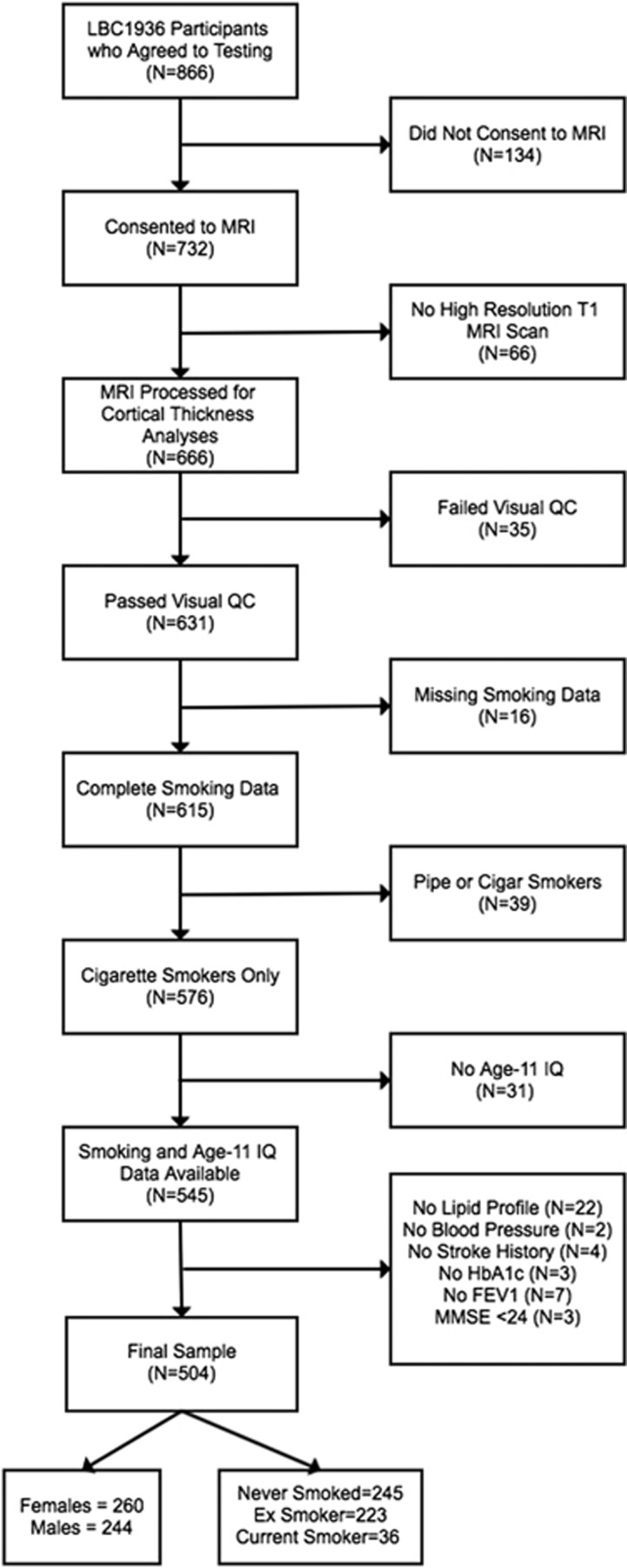
Flowchart depicting the step-by-step selection process of the Lothian Birth Cohort 1936 (LBC1936) subjects included in the final sample for data analyses. MRI, magnetic resonance imaging; IQ, intellectual quotient; FEV1, forced expiratory volume in 1 s; MMSE, mini-mental status examination; QC, quality control.

From the 666 subjects with brain MRI data, 504 (260 females) had the relevant smoking and confounding variable data available, and had no evidence of dementia (as assessed by self-report during medical history taking and by excluding subjects with a Mini Mental State Examination^20^ score below 24 (refs. 21, 22)). There were 36 current smokers, 223 ex-smokers and 245 participants with no history of smoking. Their mean age (±s.d.) was 72.7 years (±8.9 months). Their mean pack-years was 29.7 and ranged from 0.08 to 164. For further details on data availability and attrition, see Figure 1 and section 1 of Supplementary Information).

### Data collection details

All subjects were interviewed and tested individually by a trained psychologist and a research nurse during a visit to the Wellcome Trust Clinical Research Facility (http://www.wtcrf.ed.ac.uk), Western General Hospital, Edinburgh. This visit included cognitive and other psychological assessments, physical examinations, extensive history taking and blood analyses^14^ (see Table 1 for sample characteristics).

Disease history was obtained as part of a structured interview. Data were collected on current and past cigarette, cigar and pipe smoking. Detailed information was obtained regarding age at which subjects started to smoke, age at cessation for ex-smokers and average number of cigarettes smoked per day. Pack-years of smoking was calculated as the average number of cigarettes per day multiplied by years as a smoker, divided by 20 (cigarettes per pack). Data on typical, recent alcohol consumption were collected by asking about type, amount and frequency of alcoholic drinks. The HADS scale (Hospital Anxiety and Depression Scale)^23^ was administered to assess recent mood states.

Adult occupational social class, as determined from Her Majesty’s Stationery Office Classification of Occupations,^24^ was used to estimate socioeconomic status (SES). This was derived from each participant’s highest reported occupation and classified into one of six categories ranging from I (professional occupations) to V (unskilled occupations), with III divided into IIIN (non-manual) and IIIM (manual).

Blood analyses were used to obtain lipid profiles and hemoglobin A1c levels. Spirometry was used to calculate FEV1. During the physical examination, three measures of sitting diastolic and systolic blood pressure were made.

Cognitive ability was measured in June 1947 (at ~11 years of age), using the Moray House Test No. 12. The Moray House Test cognitive ability scores have high concurrent validity with gold-standard scales of intelligence such as the Stanford–Binet in childhood^25^ and the Wechsler Scale of Intelligence-III in old age.^26^

### MRI acquisition

Participants were scanned on average 2.1 months (s.d.±1.2 months) after the cognitive and medical testing. MRI scans were performed using a GE Signa Horizon HDxt 1.5 T clinical scanner (General Electric, Milwaukee, WI, USA). The protocol included T1-, T_2_-, T_2_*-weighted and FLAIR whole-brain scans. For the T1-weighted scans, high-resolution whole-brain T_1_-weighted volume scans were acquired with a field of view of 256 × 256 mm^2^, an acquisition matrix of 192 × 192 (zero-filled to 256 × 256) and 160 contiguous 1.3-mm-thick slices yielding final voxel dimensions of 1 × 1 × 1.3 mm^3^. For further details regarding the T1-weighted MRI acquisition protocol, see section 2 of Supplementary Information. For T_2_-, T_2_*-weighted and FLAIR sequence details, see Wardlaw *et al.*^19^

### MRI processing

To determine local cortical thickness measurements for each subject, the T_1_-weighted volume scans were processed using the automated CIVET pipeline (version 1.1.12) developed at the Montreal Neurological Institute (http://www.bic.mni.mcgill.ca). For details regarding CIVET processing steps, see section 3 of Supplementary Information.

In order to be able to account for the potential impacts of subclinical cerebrovascular disease, we used FLAIR images (checking T_1_- and T_2_-weighted images when necessary) to obtain each subject’s Fazekas score, a standardized visual rating scale that provides a measure of white matter hyperintensities (WMH) load.^27^ Specifically, Fazekas scores were assessed by one of two experienced ‘board certified’ neuroradiologists as periventricular (0–3) and deep (0–3) WMH scores and then summed to get a total score out of 6.

### Statistical analyses

Statistical analyses were performed using SurfStat (www.math.mcgill.ca/keith/surfstat) for local cortical thickness analyses and with SPSS 20 (SPSS, Chicago, IL, USA) for all other analyses. All analyses were conducted while accounting for the effects of gender and exact age at scanning in days, in order to account for any remaining residual age effect. For local cortical thickness analyses, a *t*-value was calculated for the slope of the *β*-coefficient (estimated from the regression model) of the variable of interest (for example, pack-years or number of years since last cigarette depending on the analysis) at each cortical point, thereby producing a three-dimensional *t*-statistic map. A *t*-value threshold of statistical significance was established taking into account multiple comparisons via the false discovery rate (FDR). For all local cortical thickness analyses, an FDR threshold of 0.05 was used to account for multiple comparisons across the cortex. However, for Model 3, a more lenient exploratory FDR=0.20 threshold was also used to further examine the data (for an FDR=0.2 threshold, the expectation is that 80% of findings are true positives). The rationale for complementing the original FDR=0.05 results with results from this more lenient exploratory threshold is that the large number of covariates used in Model 3 may lead to false negatives via decreased sensitivity of the model due to a loss in degrees of freedom. Further, given the known strong links between cigarette smoking and FEV1,^28^ as well as with cerebrovascular disease,^1^ covarying for FEV1 and Fazekas score might substantially control for the amount of smoking and hence overcontrol for the effect of interest, namely the possible impact of smoking on the cortex.

In order to provide information regarding effect size, ranges of correlation values derived from the three-dimensional *t*-statistic maps (using *r*=*t* /√(df+*t*^2^) were also calculated.

#### Model 1

As a first step, the following general linear model was used to test the association between smoking status category (that is, smokers, ex-smokers and those who have never smoked) and cortical thickness at each vertex:

Cortical thickness=***b***_**0**_Intercept+***b***_**1**_Gender+***b***_**2**_Exact_Age_At_Scanning+***b***_**3**_Smoking-Category+error

#### Model 2

Next, in order to more effectively assess the dose–response relationship between smoking and cortical thickness, the Smoking-Category term was replaced by a Pack-Years term. This analysis, as well as those of Models 3 and 4 below, were restricted to current and ex-smokers (*n*=259):

Cortical thickness=***b***_**0**_Intercept+***b***_**1**_Gender+***b***_**2**_Exact_Age_At_Scanning+***b***_**3**_PackYears+error

#### Model 3

The dose–response association was then re-examined while controlling for potential mediating and confounding variables. These covariates included HDL (high-density lipoprotein) cholesterol, LDL (low-density lipoprotein) cholesterol, HDL cholesterol ratio, triglycerides, sitting diastolic and systolic blood pressure (mean of three measures), history of high blood pressure, history of stroke or cardiovascular disease, Fazekas score, FEV1, alcohol units per week, hemoglobin A1c, diagnosis of diabetes, depression score from HADS, age-11 IQ and SES:

Cortical thickness=***b***_**0**_Intercept+***b***_**1**_Gender+***b***_**2**_Exact_Age_At_Scanning+***b***_**3**_PackYears +***b***_**4**_HDL_Cholesterol+***b***_**5**_LDL_Cholesterol+***b***_**6**_HDL_Cholesterol_Ratio+***b***_**7**_ Triglycerides+***b***_**8**_Diastolic_Pressure+***b***_**9**_Systolic_Pressure+***b***_**10**_HighBloodPressureHistory +***b***_**11**_Stroke_History+***b***_**12**_Fazekas_Score**+*b***_**13**_Cardiovascular_Disease_History +***b***_**14**_FEV1+***b***_**15**_Weekly_Alcohol_Units+***b***_**16**_Hemoglobin_A1C+***b***_**17**_Diabetes_Diagnosis +***b***_**18**_HADS+***b***_**19**_Age-11_IQ+***b***_**20**_SES+error

To assess whether the association between smoking and cortical thickness varied as a function of the age of starting smoking, Models 2 and 3 were retested after including ‘smoking starting age’ and the ‘smoking starting age’ by ‘pack-years’ interaction term.

#### Model 4

To assess whether the potential impact of smoking on cortical thickness could be reversed, we then examined the association between pack-years-adjusted cortical thickness and number of years since smoking the last cigarette. As all subjects were scanned at essentially the same age, the rationale is that for a given amount of lifetime smoking (that is, pack-years), if the possible effect of smoking is at least partially reversible, then those who stopped smoking long ago should have a thicker cortex than those who stopped smoking more recently or are currently smoking. This analysis was conducted using the following model:

Cortical thickness=***b***_**0**_Intercept+***b***_**1**_Gender+***b***_**2**_Exact_Age_At_Scanning+**b**_**3**_Number_Of_Years_Since_Last_Cigarette+***b***_**4**_Pack-Years+error

## Results

### Demographics

The mean age (mean±s.d.) at MRI was 72.7±0.74 years for those that never smoked, 72.7±0.76 for ex-smokers and 72.7±0.63 for current smokers. The mean pack-years value was 26.9±29.15 for ex-smokers and 46.9±25.07 for current smokers. Table 1 further describes the mean, range, frequency and variance of basic demographic and clinical variables, partitioned by smoking category. Of note, there were no significant differences in age at MRI, gender distribution, SES and IQ at age 11 years between the three smoking categories (never smoked, ex-smokers and current smokers), suggesting that the groups were reasonably well matched at baseline. In fact, the three smoking categories only differed for cardiovascular disease history (*χ*^2^(2)=10.77, *P*=0.005), forced expiratory volume in 1 s (F (2501)=7.53, *P*=0.001) and alcohol units per week (F (2501)=5.11, *P*=0.006).

### Associations between smoking status and cortical thickness

**Figure 2:**
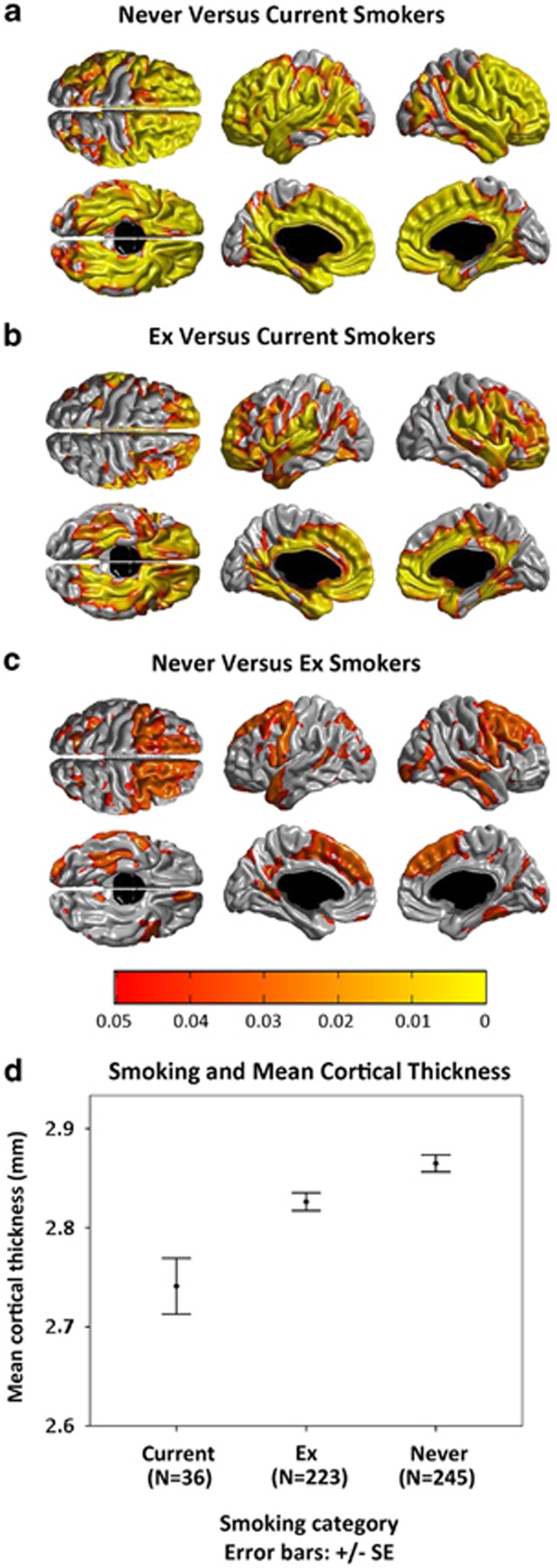
Cortical thickness contrasts between the three smoking categories. Areas in orange–yellow shades represent statistically significant group differences at a falsediscovery rate (FDR)=0.05. Color bar represents FDR *q-*values. For each panel, the top left brain image represents a view from the top, the bottom left image represents a view from the bottom (the black section is where the brain stem begins), the middle top image represents the left lateral view, the middle bottom image represents the left medial view (the black section is where the hemispheres meet), the top right image represents the right lateral view and the bottom right image represents the right medial view. (**a**) Areas in which those that never smoked have a thicker cortex than current smokers. (**b**) Areas in which ex-smokers have a thicker cortex than current smokers. (**c**) Areas in which those that never smoked have a thicker cortex than ex-smokers. (**d**) Mean cortical thickness ±1s.e.m. of current, ex- and those that never smoked.

Controlling for gender and exact age at scanning (Model 1) showed that current smokers had a generally thinner cortex than those who had never smoked (Figure 2a). Many of the most significant regions of associations were in prefrontal areas. Nonetheless, associations were widespread, including most of the cortex and sparing only some primary motor/sensory and occipital areas. Using the same model but comparing ex-smokers with current smokers revealed a similar but less extensive pattern of thinner cortex for current smokers (Figure 2b). Relatively thinner cortex was predominantly located in large areas of the medial and lateral frontal cortex, in addition to some regions of the medial and lateral temporal and parietal cortices. Comparing ex-smokers with participants who had never smoked also showed a thinner cortex for ex-smokers in many areas (Figure 2c). Associations were less extensive than for the previous two contrasts, but peaks were, here also, predominantly located in prefrontal areas.

Using the same model as above (Model 1), a highly significant association between smoking status and mean cortical thickness was observed (F (2, 499)=14.7, *P*<0.001; Figure 2d). Follow-up analyses on this model showed that all pairwise comparisons between the three smoking categories were significant (Bonferroni-corrected threshold of *P*⩽0.017; for details, including Cohen’s *d*-values, see Supplementary Table S1).

### Dose-dependent association between smoking and cortical thickness

**Figure 3:**
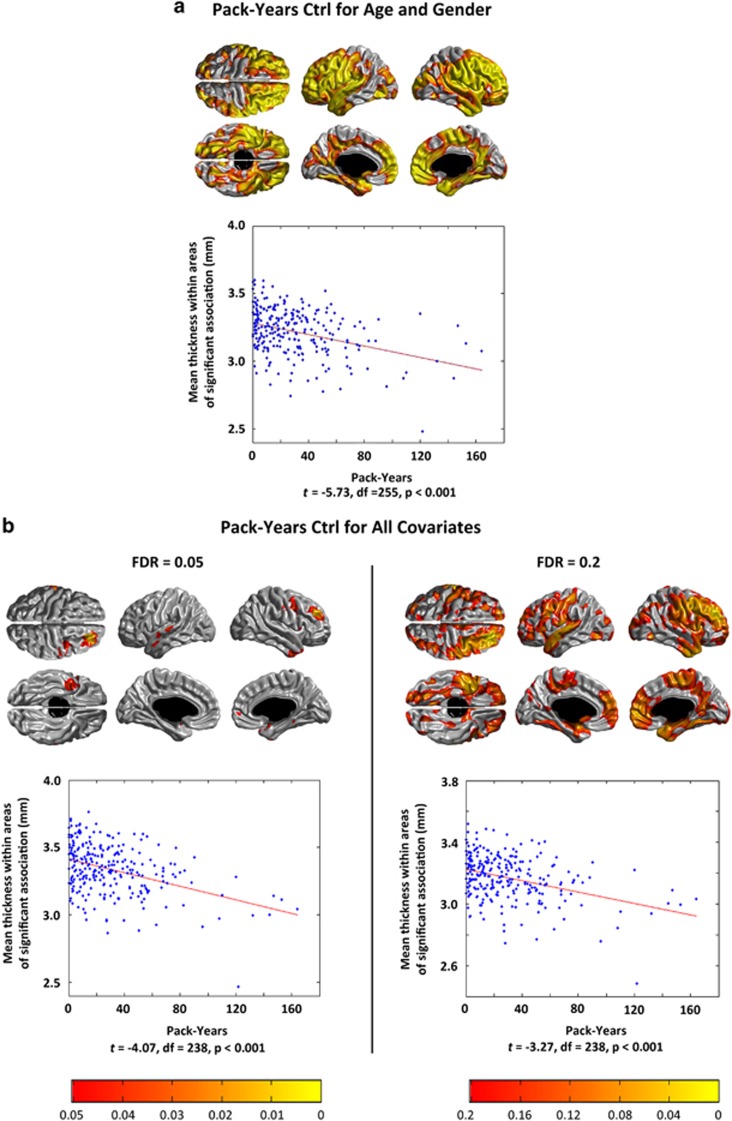
Associations between the number of pack-years and local cortical thickness (*n*=259). Areas in orange–yellow shades represent statistically significant associations at a false discovery rate (FDR)=0.05. Color bar represents FDR *q-*values. For a description of what three-dimensional (3D) perspective each specific brain image represents, see Figure 2 legend. (**a**) Association between pack-years and cortical thickness controlling only for gender and exact age at scanning. A scatterplot of the association between pack-years and mean cortical thickness within significant areas of thinning is also provided (thickness values in mm). (**b**) Association between pack-years and cortical thickness controlling for all covariates. A scatterplot of the association between pack-years and mean cortical thickness within significant areas of thinning is also provided (thickness values in mm).

Controlling for gender and exact age at scanning (Model 2) in current and ex-smokers (*n*=259) showed negative widespread associations between pack-years and cortical thickness (Figure 3a). Within areas of significant associations, partial Pearson correlation values ranged from −0.35 to −0.11 (mean±s.d.; −0.18±0.04). Associations had a distribution (Supplementary Table S2) similar to the one observed in the categorical analyses done above using Model 1.

Re-examining the dose–response smoking association after controlling for potential mediating and confounding variables (Model 3) with an FDR threshold set at 0.05 revealed a strongly attenuated pattern of significant associations. Nonetheless, peaks were mainly located in prefrontal areas here as well. Within areas of associations, the partial Pearson correlation values ranged from −0.29 to −0.19 (mean±s.d.; −0.22±0.02). Using the more lenient FDR=0.2 threshold uncovered the original pattern of association found in Figure 3a, where only gender and exact age at scanning were used as covariates. Within areas of associations, the partial Pearson correlation values ranged from −0.29 to −0.09 (mean±s.d.; −0.13±0.03). Here too, peaks were mainly located in prefrontal areas.

A significant association between pack-years and global mean cortical thickness was observed when controlling for only gender and exact age at scanning (Model 2; F (1, 255)=18.9, *P*<0.001, partial Pearson correlation=−0.26; see Figure 3a) and when also controlling for all covariates (Model 3; F (1, 238)=4.5, *P*=0.035, partial Pearson correlation=−0.14 ;see Figure 3b).

No interactions between starting age and pack-years were found in any analyses.

*Post-hoc* explorations revealed that the greatest attenuation in number of significant vertices for the association between cigarette smoking and cortical thickness was due to the inclusion of FEV1 and Fazekas score in the model (see Supplementary Figure S1).

### Reversibility of the smoking–cortical thickness association

**Figure 4:**
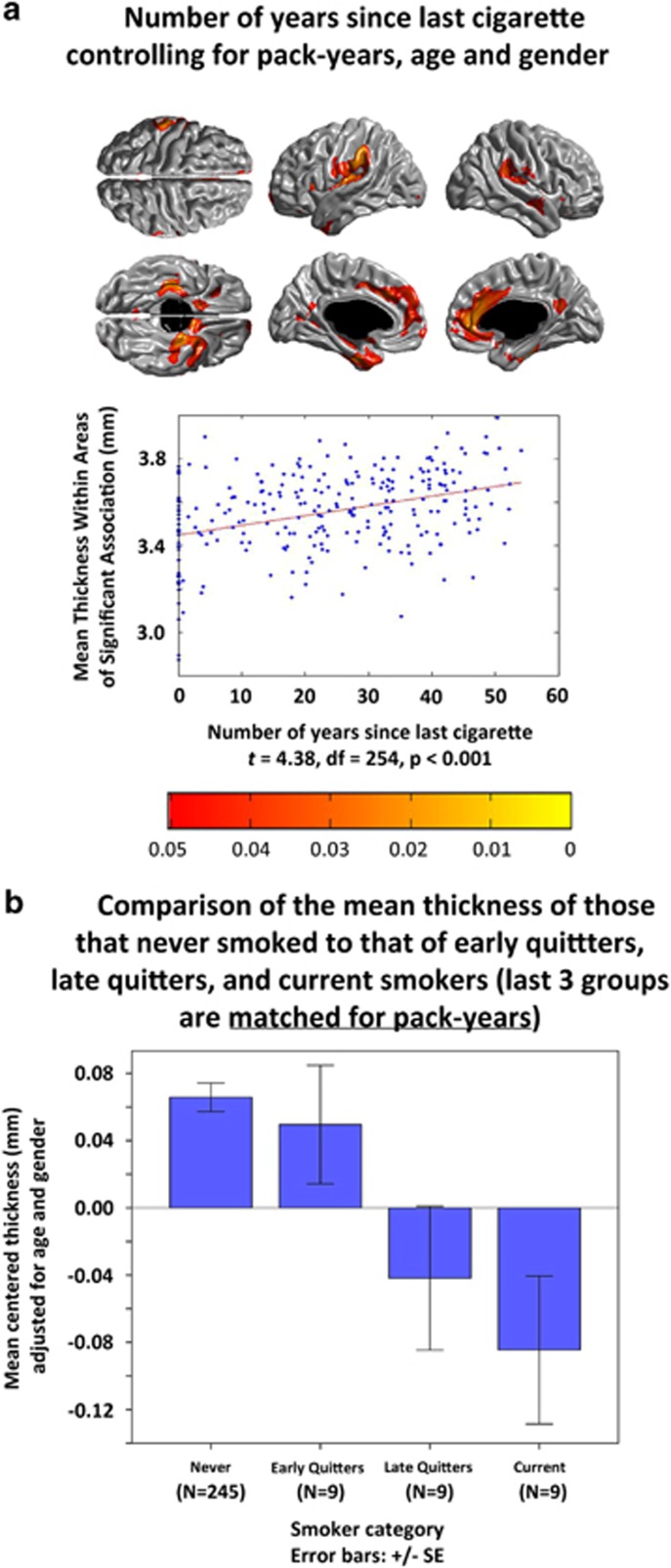
Associations between the number of years since last smoked and local cortical thickness (*n*=259). Areas in orange–yellow shades represent statistically significant associations at a false discovery rate (FDR)=0.05. Color bar represents FDR *q-*values. For a description of what three-dimensional (3D) perspective each specific brain image represents, see Figure 2 legend. (**a**) Association between the number of years since last smoked and cortical thickness controlling for pack-years, gender and exact age at scanning. A scatterplot of the association between pack-years and mean cortical thickness within significant areas of recovery is also provided (thickness values in mm). (**b**) For visualization purposes, a bar graph depicting centered mean cortical thickness within areas of recovery in a sub-sample of early quitters (⩾28 years since quitting), late quitters (<28 years since quitting) and current smokers precisely matched for pack-years. The mean pack-years for each of the three groups is 24.4 pack-years. The mean reported number of cigarettes smoked per day was 8.9 for current smokers against 16.1 for late quitters and 31.7 cigarettes for early quitters. The complete subsample of those that never smoked was used in order to get the best possible estimate of mean thickness for that group, as it was not constrained by the requirements of matching for pack-years.

Controlling for pack-years, gender and exact age at scanning (Model 4), positive associations were shown between cortical thickness at age 73 years and number of years since smoking the last cigarette (Figure 4a and Supplementary Table S3). We shall name these significant regions as areas of ‘recovery,’ yet acknowledge that although supported, this labeling is not derived from longitudinal data. Peaks, which had all been shown in the association with smoking in the above analyses using Models 1 and 2, were mainly located, bilaterally, in the supramarginal gyrus, the superior temporal gyrus, the anterior and posterior cingulate, the posterior insular cortex and the fusiform and parahippocampal gyri. For a plot, within regions of recovery, of years since quitting against mean cortical thickness adjusted for pack-years, gender and exact age, see Figure 4a. The rate, within these regions, of mean cortical thickness recovery adjusted for pack-years, gender and exact age at scanning was 3.69 μm per year since the last cigarette (95% confidence interval, 2.03 to 5.35 μm; *P*<0.001). Conversely, adjusting for gender and exact age at scanning, but looking instead at rate of thinning in current smokers (to avoid confounding this rate by the effect of recovery that is observed in ex-smokers), revealed, within areas of pack-years-associated cortical thinning, that mean cortical thickness was 3.21 μm thinner for every pack-year smoked (95% confidence interval, −5.82 to −0.60 μm; *P*=0.017). This suggests that it takes ~0.9 years without smoking, to recover from the possible cortical thinning effect of each pack-year. On this basis and given that the average pack-years in this sample’s current and ex-smokers was 29.7, it took roughly 25 years without smoking for differences in cortical thickness to no longer be observed between ex-smokers and those that never smoked. However, heavy ex-smokers remained with a thinner cortex at age 73 years even after more than 25 years without smoking.

Conclusions regarding the potential reversibility of the possible impact of smoking on the cortex should mainly be based on Model 4 results as well as associated Figure 4a. However, to help visualization, three groups of smokers (current smokers, late quitters and early quitters) that matched exactly for pack-years (24.4 pack-years) were created. The median value of time since quitting (28 years) was used as cutoff to distinguish ‘early’ and ‘late’ quitters. The group with the greatest mean thickness was the group that never smoked, followed by the early quitters, the late quitters and the current smokers, respectively (see Figure 4b). Note that the last three groups have smoked the exact same amount during their lifetime.

## Discussion

This study demonstrates a dose-dependent negative association between cigarette smoking and cortical thickness that is more extensive than previously reported.^4, 5, 6, 7, 10, 11, 12^ Although for each pack-year smoked, the cortex is only very slightly thinner than in subjects who never smoked, this rate of smoking-related thinning is approximately twice that of a previously observed yearly rate of mean cortical thinning in typical adult populations.^29^ This being said, most smokers in this sample smoked much more than one pack-year and tended to have a cortical thickness compatible with an even greater rate of thinning. The other key finding is a positive association between number of years without smoking and cortical thickness after accounting for the lifetime ‘dose’ of smoking; this result supports a reversal, after smoking cessation, of the potential impact of smoking on the cortex.

Before the current work, the largest study that had looked at associations between smoking and cortical thickness had a sample size of 118 subjects^11^ (that is, almost five times less than the current study) with only 76 of these 118 subjects actually used to examine the effect of smoking. In all likelihood, it is the combination of the statistical power of the large brain-imaging sample of the LBC1936 and of the use of the cortical thickness metric that made it possible to discover such a widespread negative association between cigarette smoking and cortical tissue. Such a diffuse association is in keeping with reported negative associations between smoking and cognitive ability shown in the LBC1936 (ref. 2) and in other samples.^30^ Associations between pack-years and cortical thinning were greatest in statistical significance in the medial and lateral prefrontal areas, posterior cingulate gyrus, precuneus, medial and lateral temporal areas, and the angular gyrus, regions that are all part of the default mode network.^31^ Given the known breakdown in functional connectivity of the default mode network in Alzheimer’s disease, these observations accord with reported associations between smoking and increased risk of Alzheimer’s disease.^3^

Findings here suggest that cortical recovery could take as little as a few weeks to more than a theoretical 140 years (mean of ~25 years for the LBC1936), depending on the amount smoked over one’s lifetime. Although the mechanisms behind the observed potential recovery of cortical thickness after smoking cessation are unknown, plausible and potentially complementary models of the dynamics of recovery can nonetheless be proposed. Although, in adulthood, the cortex tends to thin slowly with age,^29^ findings here suggest that cigarette smoking might accelerate this process. Within this framework, one possibility could be that after smoking cessation, the rate of cortical thinning decreases to such an extent that it becomes slower than that seen in normal aging non-smoking adults, leading to an eventual ‘catching up’ of ex-smokers. An alternative, of course, is that smoking cessation is associated with some degree of cortical thickening. Both proposed mechanisms are compatible with a homeostatic set point view of cortical thickness where the cortex would tend to settle towards its ‘normal age’ set point after smoking cessation. Having said this, the imputed reversal was not observed in all regions where there was a negative association between smoking and cortical thickness. This is not surprising given that smoking is likely associated to some degree of microvascular-related lesions with irreversible secondary cortical loss.^32^

Controlling for all covariates (Model 3) showed a weaker but nonetheless persistent negative association between smoking and cortical thickness, which is compatible with part of the association being due to the direct neurotoxic effects of cigarette smoking. Although to establish this completely would require more mechanistic evidence, a direct neurotoxic effect of cigarette smoking is compatible with reported associations in rodents between prenatal exposure to nicotine and reduced cingulate cortex volume, dopamine turnover, brain size, dendritic morphology and spine density.^33, 34^

Conversely, the attenuation of the association between smoking and cortical thickness, when controlling for all covariates suggests that some of the possible impact of cigarette smoking on the cortex is indirect. The five covariates with the greatest impact all had an attenuating effect on results. They include FEV1, Fazekas score, IQ, HADS score and SES (for details, see Supplementary Figure S1). Of all the covariates, FEV1 had the strongest impact on results, a finding compatible with links between chronic obstructive pulmonary disease, chronic hypoxemia and cognitive impairment.^35^ Fazekas score had the second strongest attenuating influence on results, suggesting that subclinical vascular disease secondary to cigarette smoking might also have a role in the association between smoking and cortical changes. However, as noted in the methods, given known strong links between cigarette smoking and FEV1 (ref. 28) as well as with cerebrovascular disease,^1^ it is necessary to acknowledge the possibility that covarying for FEV1 and Fazekas score might substantially control for the amount of smoking and hence overcorrect for the possible impact of smoking on the cortex.

A limitation of this study is its observational nature. Further, one needs to be cognizant of the possibility that reverse causation may account for a certain amount of the association between smoking and cortical thinning. For instance, we show that IQ at 11 years accounts for a certain degree of association between smoking and cortical thickness (Supplementary Figure S1). This supports the view that a certain proportion of the cortical thickness/smoking association is not due to direct or indirect effects of cigarette smoking on the cortex given that those with a lower IQ have both a thinner cortex^16, 36^ and a higher propensity to smoke.^2^ Similarly, structural differences in cortical areas related to impulse control (for example, the orbitofrontal cortex) might predispose to smoking and therefore predate it rather than follow from it.^37^ This being said, the identified dose-dependent associations in diffuse brain areas (including regions without evidence for a role in impulse control) and the apparent reversal of the effect of smoking on the cortex provide evidence that the differences in thickness do not mostly predate the onset of smoking. In addition, although the sample of current smokers (*n*=36) is greater than most studies looking at the impact of smoking on the brain, it remains a relatively small sample. Another limitation is the recalled nature of the smoking history data; however, recall errors would tend to add noise to the data and reduce the degree of association between smoking and cortical thickness. On such a basis, the degree of association between smoking and cortical thickness is likely to be even stronger than observed here. This being said, none of these caveats have an impact on the conclusions of the current work or decrease many of its strengths, which include the following: blinded quality control of imaging data; having all participants scanned at the same age, thereby minimizing potential confounding complex age effects; having the largest sample to date examining smoking-related cortical thickness associations; and availability of cognitive ability before smoking, which provided the rare opportunity to rule out a potential important confounder to smoking/brain structure associations.

Smokers need to be informed that cigarettes are associated with accelerated cortical thinning, a biomarker of cognitive aging. Importantly, cortical thinning can persist for many years after smoking cessation. The potential to at least partially recover from smoking-related thinning might serve as a strong motivational argument to encourage smoking cessation.

## Figures and Tables

**Table 1:** Detailed demographic variables and covariates divided by smoking categories

	*Never smoked* n=*245*	*Ex-smokers* n=*223*	*Current smokers* n=*36*
Age at MRI (years)	72.7±0.7 (71.2–74.2)	72.7±0.8 (71.0–74.2)	72.7±0.6 (71.6–73.9)
			
Gender	Female=136 (55.5)	Female=104 (46.6)	Female=20 (55.6)
	Male=109 (44.4)	Male=119 (53.4)	Male=16 (44.4)
			
IQ at age 11 years	101.6±15.2 (38.5–128.0)	101.8±14.3 (62.1–129.9)	99.8±10.7 (78.3–119.6)
			
Socioeconomic status	I=58 (23.7)	I=47 (21.1)	I=3 (8.3)
	II=90 (36.7)	II=94 (42.2)	II=7 (19.4)
	IIIN=57 (23.3)	IIIN=34 (15.2)	IIIN=15 (41.7)
	IIIM=35 (14.3)	IIIM=38 (17.0)	IIIM=11 (30.6)
	IV=6 (1.6)	IV=9 (4.0)	IV=0 (0)
	V=1 (0.4)	V=1 (0.4)	V=0 (0)
			
Cardiovascular disease	No=195 (79.6)	No=149 (66.8)	No=29 (80.6)
History^a^	Yes=50 (20.4)	Yes=74 (33.2)	Yes=7 (19.4)
Cerebrovascular	No=203 (82.9)	No=189 (84.8)	No=26 (72.2)
Accident history	Yes=42 (17.1)	Yes=34 (15.2)	Yes=10 (27.8)
Mean systolic blood pressure (mm Hg)	148.9±19.0 (98.3–217.0)	149.8±17.5 (98.3–198.7)	146.3±20.3 (82.3–188)
Mean diastolic blood pressure (mm Hg)	78.6±9.3 (56.3–103.0)	77.2±9.3 (50.7–104.7)	77.6±11.1 (50.7–103.7)
Total cholesterol (mmol l^−1^)	5.3±1.1 (2.5–9.2)	5.1±1.5 (2.8–10.1)	5.4±1.2 (3.0–7.6)
LDL (mmol l^−1^)	3.1±1.0 (0.8–5.9)	2.9±1.1 (0.6–7.5)	3.1±1.3 (0.5–5.5)
HDL (mmol l^−1^)	1.5±0.4 (0.7–2.9)	1.4±0.4 (0.7–2.8)	1.6±0.5 (0.9–3.5)
Cholesterol/HDL ratio	3.7±1.1 (1.7–7.7)	3.7±1.1 (1.4–7.4)	3.8±1.3 (1.3–6.1)
Triglycerides (mmol l^−1^)	1.5±0.8 (0.4–4.5)	1.7±0.8 (0.4–4.3)	1.7±0.7 (0.6–3.1)
Diabetes history	No=224 (91.4)	No=193 (86.5)	No=34 (94.4)
	Yes=21 (8.6)	Yes=30 (13.5)	Yes=2 (5.6)
			
HbA1c (%)	5.7±0.6 (4.4–8.6)	5.8±0.7 (4.8–9.1)	5.7±0.5 (4.6–7.2)
Forced expiratory volume (l)^b^	2.4±0.7 (0.4–4.2)	2.3±0.6 (0.8–3.9)	1.9±0.7 (0.8–3.25)
Hospital anxiety and depression scale score	2.3±2.0 (0–11)	2.7±2.2 (0–12)	2.9±2.2 (0–9)
Alcohol units per week^c^	8.1±11.8 (0–84)	11.9±13.5 (0–63)	12.4±23.6 (0–120)

Abbrevations: ANOVA, analysis of variance; HDL, high-density lipoproyein; LDL, low-density lipoprotein; MRI, magnetic resonance imaging.

Results for continuous variables are reported as mean±s.d. (range).

Results for categorical variables are reported as *n* (percentage).

There are no statistically significant differences between groups, except for the three following variables:

a Cardiovascular disease history: *χ*^2^(2)=10.77, *P*=0.005; only contrast between subject that never smoked and ex-smoker is significant with Fisher’s exact test (*P*=0.002).

b Forced expiratory volume in 1 s: one-way ANOVA F (2501)=7.53, *P*=0.001; never versus current and ex versus current smoker contrasts significant with *post-hoc* Tukey’s test.

c Alcohol units per week: one-way ANOVA F (2501)=5.11, *P*=0.006; only never versus ex-smoker contrast significant with *post-hoc* Tukey’s test.
