# Supplementary material for: Cigarette smoking and thinning of the brain's cortex
Source: Mol Psychiatry. 2015 Feb 10;20(6):778–85. doi: 10.1038/mp.2014.187 (PMC4430302; doi:10.1038/mp.2014.187)
Supplement: Supplementary Information [file mp2014187x1.doc]

**SUPPLEMENTARY INFORMATION**

**1) Sample Attrition Details**

From the set of 666 with structural brain imaging of sufficient resolution, thirty-five participants failed visual quality control (QC) of the gray or white matter surfaces (see below for further details on the cortical thickness pipeline). From the remaining 631 participants, 16 had missing smoking data and a further 39 had a history of pipe or cigar smoking, leaving a sample of 576. From these 576, 31 had no age-11 IQ data and 3 more were excluded because of a Mini Mental Status Examination score of less than 24, possibly indicating dementia. From the remaining 542 participants, 38 had missing data on other potential confounding or mediating variables (see statistical analyses section below for a list of these variables). This left a final sample of 504 participants (260 females) with a mean age (for attrition flow chart, see Figure 1). This sample included 36 current smokers, 223 ex-smokers, and 245 participants with no history of smoking.

**2) MRI Acquisition Protocol**

The GE Signa Horizon HDxt 1.5 T clinical scanner (General Electric, Milwaukee, WI, USA) used here was equipped with a self-shielding gradient set (33 mT m^−1^ maximum gradient strength) and manufacturer supplied eight-channel phased-array head coil. Repetition, echo and inversion times were 10, 4 and 500 ms, respectively. In order to ensure total brain coverage, slices were carefully placed to cover the complete contents from above the skull vertex to the upper cervical spine below the foramen magnum.

**3) MRI Processing**

The CIVET pipeline steps include: (1) registering T_1_W images to a standardized space using an age-specific template for the population under study; (2) correcting for intensity non-uniformity artifacts (bias field); (3) producing high-resolution hemispheric surfaces with 40962 vertices each; (4) registering surfaces to a high-resolution template to establish inter-subject correspondence of vertices; (5) applying a reverse of step 1 to allow cortical thickness estimations in the native space of each subject; (6) calculating cortical thickness at each vertex using the t-link metric; and (7) smoothing using a 20-mm kernel.

Importantly, a visual quality control of the native cortical gray and white matter surfaces was performed blinded to each participant’s characteristics including smoking status. The goal of this process was to ensure that there were no significant aberrations in cortical thickness estimations for any subject. Scans with clear problems in the cortical thickness maps because of ringing or other artifacts (mainly caused by movement in the scanner) were eliminated from statistical analyses.

**4) Supplementary Figure and Table Legends**

**Figure S1. Flowchart of the impact of the inclusions of the covariates on the association between pack-years and cortical thickness.** All analyses are controlled for exact age at scanning and gender. For each analysis, the total number of significant vertices and percentage of significant vertices lost compared to the basic pack-years analysis (top) are included in the boxes. Figures of the association between pack-years controlling for age and gender only (top), and controlling for all covariates (bottom) are provided. Areas in orange-yellow shades represent statistically significant associations at FDR=0.05. Color bar represents FDR *q* values*.* For a description of what 3D perspective each specific brain image represents, see Figure 2 legend.

**Table S1. Global mean thickness comparison between the three smoking categories.** Note that the distribution of global mean cortical thickness followed a normal distribution (Mean ± SD; 2.84 mm ± 0.14).

**Table S2. List of the 10 anatomical areas with the most significant peak vertices for the general linear model of pack-years against cortical thickness, controlling for age at scanning, and gender.** MNI coordinates of the peak vertex within each anatomical area are provided for reference purpose. The ten areas containing the most significant peaks were selected, but only one vertex was selected for each anatomical region (e.g., many peaks in the right rostral middle frontal were more significant than the most significant peak in the right temporal pole, but only the most significant one is provided). P-values corrected for multiple comparisons with vertex-wide random field theory are provided at the peak level, and at the cluster level. The Desikan surface atlas[^1^](#_ENREF_1) was used to label anatomical areas.

**Table S3.** **List of the anatomical areas with significant peak vertices for the general linear model of number of years since last smoked against cortical thickness, controlled for pack-years, age at scanning, and gender.** MNI coordinates of the peak vertex within each anatomical area are provided for reference purpose. Only the most significant vertex was selected for each anatomical region. P-values corrected for multiple comparisons with vertex-wide random field theory are provided at the peak level, and at the cluster level. The Desikan surface atlas[^1^](#_ENREF_1) was used to label anatomical areas.


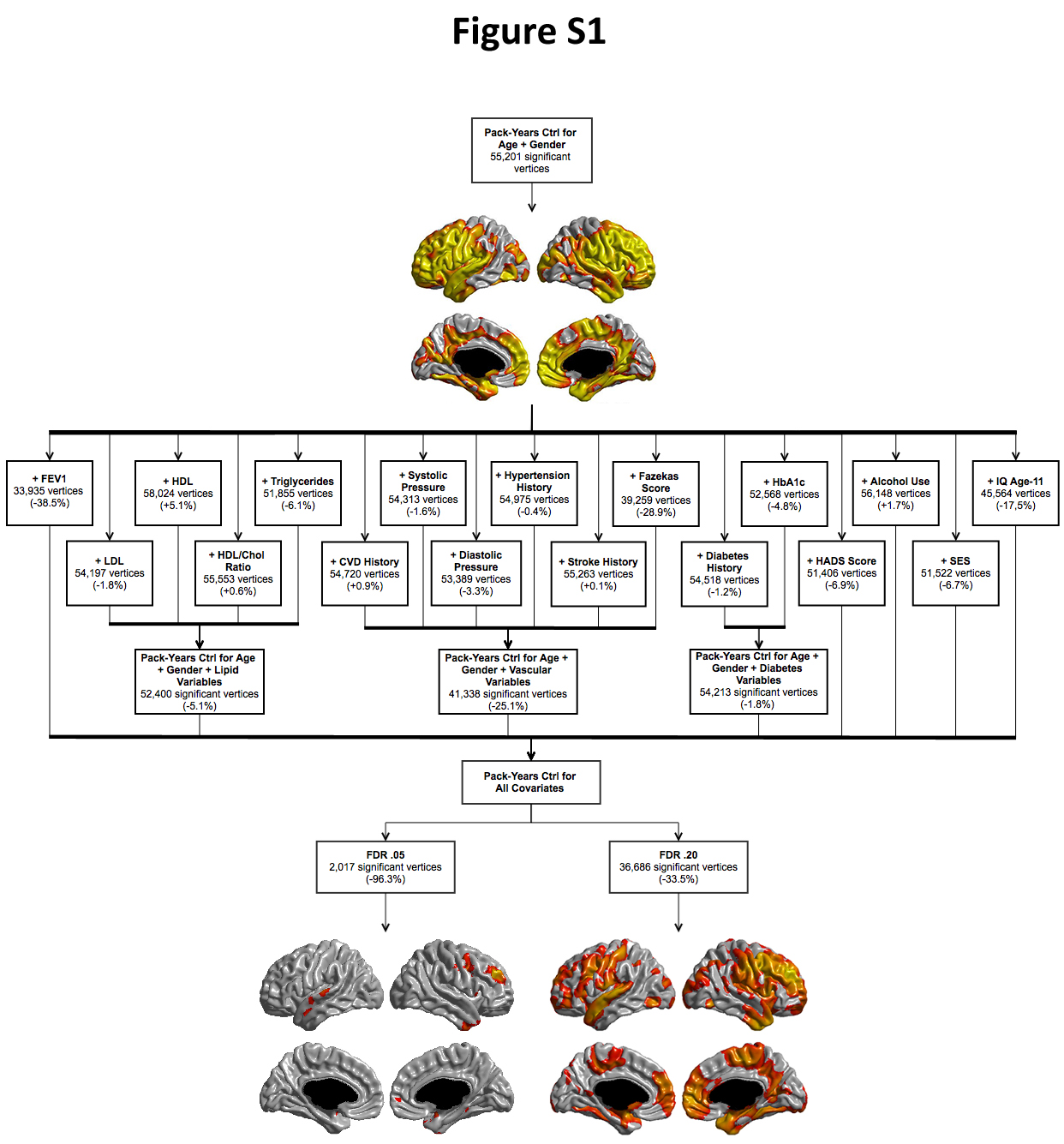


**TABLE S1**

Global mean thickness comparison between the three smoking categories

| Category | *F* statistic | *P* value | Cohen's d |
| --- | --- | --- | --- |
| Ex-smokers versus current smokers | F(1,266) = 14.76 | P<0.001 | 0.56 |
| Ex-smokers versus subjects that never smoked | F(1,488) = 7.12 | P<0.008 | 0.29 |
| Subjects that never smoker versus current smokers | F(1,302) = 28.20 | P<0.001 | 0.81 |

**TABLE S2**

| **Anatomical Area** | **Peak Coordinates** | **T Score** | **Corrected**  **P-value**  **(peak level)** | **Corrected**  **P-value**  **(cluster level)** |
| --- | --- | --- | --- | --- |
| **Right Rostral Middle Frontal** | x = 23.0  y = 44.4  z = 35.8 | 5.908 | <0.001 | <0.001 |
| **Left Superior Temporal** | x = -60.3  y = -19.6  z = 7.9 | 5.5037 | <0.001 | <0.001 |
| **Right Superior Frontal** | x = 17.9  y = 43.5  z = 38.6 | 5.076 | 0.001 | <0.001 |
| **Right Caudal Frontal** | x = 40.1  y = 2.8  z = 41.6 | 5.01 | 0.002 | <0.001 |
| **Right Enthorinal** | x = 30.7  y = 4.5  z = -18.2 | 4.8051 | 0.004 | <0.001 |
| **Right Temporal Pole** | x = 42.1  y = 2.3  z = -45.4 | 4.7587 | 0.005 | <0.001 |
| **Right Fusiform** | x = 38.9  y = 3.8  z = -47.1 | 4.7424 | 0.005 | <0.001 |
| **Right Medial Orbitofrontal** | x = 9.6  y = 55.8  z = -1.1 | 4.5531 | 0.011 | <0.001 |
| **Left Post Central** | x = -62.3  y = -16.6  z = 17.7 | 4.5265 | 0.013 | <0.001 |
| **Right Cuneus** | x = 5.4  y = -58.2  z = 14.4 | 4.2799 | 0.032 | <0.001 |

**TABLE S3**

| **Anatomical Area** | **Peak Coordinates** | **T Score** | **Corrected**  **P-value**  **(peak level)** | **Corrected**  **P-value (cluster level)** |
| --- | --- | --- | --- | --- |
| **Left Fusiform** | x = -29.5  y = -9.0  z = -33.2 | 4.5093 | 0.014 | 0.044 |
| **Left Supra Marginal** | x = -62.7  y = -31.7  z = 35.5 | 4.3682 | 0.023 | 0.021 |
| **Left Insula Pars Orbicularis** | x = -40.4  y = -11.3  z = 18.0 | 4.1538 | 0.052 | 0.001 |
| **Right Fusiform** | x = 35.1  y = -22.3  z = -26.8 | 3.9647 | 0.102 | 0.016 |
| **Right Rostral Anterior Cingulate** | x = 6.4  y = 41.8  z = 4.4 | 3.9611 | 0.103 | <0.001 |

1. Desikan RS, Segonne F, Fischl B, Quinn BT, Dickerson BC, Blacker D *et al.* An automated labeling system for subdividing the human cerebral cortex on MRI scans into gyral based regions of interest. *NeuroImage* 2006; **31**(3)**:** 968-980.
